# A comprehensive evaluation of the immune system response and type-I Interferon signaling pathway in hospitalized COVID-19 patients

**DOI:** 10.1186/s12964-022-00903-6

**Published:** 2022-07-16

**Authors:** Mohammad Sadegh Soltani-Zangbar, Forough Parhizkar, Elham Ghaedi, Ali Tarbiat, Roza Motavalli, Amin Alizadegan, Leili Aghebati-Maleki, Davoud Rostamzadeh, Yousef Yousefzadeh, Golamreza Jadideslam, Sima Shahmohammadi Farid, Leila Roshangar, Ata Mahmoodpoor, Javad Ahmadian Heris, Abolfazl Miahipour, Mehdi Yousefi

**Affiliations:** 1 Student Research Committee, Tabriz University of Medical Sciences, Tabriz, Iran; 2 Stem Cell Research Center, Tabriz University of Medical Sciences, Tabriz, Iran; 3 Department of Immunology, School of Medicine, Tabriz University of Medical Sciences, Tabriz, Iran; 4 Pirogov Russian National Research Medical University, Moscow, Russia; 5 Department of Cardiology, Medical Faculty, Urmia University of Medical Sciences, Urmia, Iran; 6 Department of Reproductive Biology, Faculty of Advanced Medical Sciences, Tabriz University of Medical Sciences, Tabriz, Iran; 7 Immunology Research Center, Tabriz University of Medical Sciences, Tabriz, Iran; 8 Medicinal Plants Research Center, Yasuj University of Medical Sciences, Yasuj, Iran; 9 Department of Molecular Medicine, Faculty of Advanced Medical Sciences, Tabriz University of Medical Sciences, Tabriz, Iran; 10 Department of Anesthesiology, Faculty of Medicine, Tabriz University of Medical Sciences, Tabriz, Iran; 11 Department of Allergy and Clinical Immunology, Pediatric Hospital, Tabriz University of Medical Sciences, Tabriz, Iran; 12 Department of Parasitology and Mycology, School of Medicine, Alborz University of Medical Sciences, Karaj, Iran

**Keywords:** Immune-phenotype, COVID-19, IFN-I, Signaling pathway, Illness severity

## Abstract

**Background:**

The COVID-19 pandemic has become the world’s main life-threatening challenge in the third decade of the twenty-first century. Numerous studies have been conducted on SARS-CoV2 virus structure and pathogenesis to find reliable treatments and vaccines. The present study aimed to evaluate the immune-phenotype and IFN-I signaling pathways of COVID-19 patients with mild and severe conditions.

**Material and methods:**

A total of 100 COVID-19 patients (50 with mild and 50 with severe conditions) were enrolled in this study. The frequency of CD4 + T, CD8 + T, Th17, Treg, and B lymphocytes beside NK cells was evaluated using flow cytometry. IFN-I downstream signaling molecules, including JAK-1, TYK-2, STAT-1, and STAT-2, and Interferon regulatory factors (IRF) 3 and 7 expressions at RNA and protein status were investigated using real-time PCR and western blotting techniques, respectively. Immune levels of cytokines (e.g., IL-1β, IL-6, IL-17, TNF-α, IL-2R, IL-10, IFN-α, and IFN-β) and the existence of anti-IFN-α autoantibodies were evaluated via enzyme-linked immunosorbent assay (ELISA).

**Results:**

Immune-phenotyping results showed a significant decrease in the absolute count of NK cells, CD4 + T, CD8 + T, and B lymphocytes in COVID-19 patients. The frequency of Th17 and Treg cells showed a remarkable increase and decrease, respectively. All signaling molecules of the IFN-I downstream pathway and IRFs (i.e., JAK-1, TYK-2, STAT-1, STAT-2, IRF-3, and IRF-7) showed very reduced expression levels in COVID-19 patients with the severe condition compared to healthy individuals at both RNA and protein levels. Of 50 patients with severe conditions, 14 had anti-IFN-α autoantibodies in sera. Meanwhile, this result was 2 and 0 for patients with mild symptoms and healthy controls, respectively.

**Conclusion:**

Our results indicate a positive association of the existence of anti-IFN-α autoantibodies and immune cells dysregulation with the severity of illness in COVID-19 patients. However, comprehensive studies are necessary to find out more about this context.

**Video abstract**

**Supplementary Information:**

The online version contains supplementary material available at 10.1186/s12964-022-00903-6.

## Background

COVID-19 is a pandemic virus that has been released worldwide since December 2019. It is also known as SARS-COV-2 due to its SARS-COV-like properties and belongs to beta viruses [1]. The manifestations of COVID-19 range from no symptoms to severe clinical tolls, like acute respiratory distress syndrome (ARDS) or death. Until now, no efficient therapeutic approach has been discovered for severe COVID-19 cases. Therefore, the regions affected by this virus cannot efficiently control the prevalence of the disease [2, 3]. Belonging to the order Nidoviride, these microorganisms are divided into four families: alpha, beta, gamma, and delta [4]. These medium-sized (approximately 120 nm) and enveloped viruses have single-stranded RNA 27–32 bases. Among this family of viruses, some of them are not pathogenic for humans, and most of them are associated with mild clinical symptoms. Two viruses of this family (i.e., SARS and MERS) have caused severe clinical symptoms in patients [5, 6]. The epidemic of SARS-COV transpired in November of 2002 in Guangdong, southern China, caused approximately 8,000 infections in humans and 774 mortality in 37 countries during 2002 to 2003. Another similar epidemic, i.e., “MERS-COV”, was first discovered in Saudi Arabia in 2012, resulting in 2,944 confirmed infections and 858 deaths worldwide [4].

In viral infections, immune responses efficiency hinged on the effective activation of T cells. Accordingly, effective T cell function with proper populations is critical for better recovery in patients with severe COVID-19 [7]. Cytotoxic CD8 + T (CTL) cells kill cells infected with the virus by producing various molecules such as perforin and granzymes as antiviral mongers [8]. Also, CD4 + helper T (Th) cells help CTLs and B Lymphocytes by secreting cytokines to expand their pathogen clearance ability [9, 10]. However, T cells overexpress inhibitory molecules such as programmed death 1 (PD-1) in long-term viral infections. As a result, they prevent the effects of virus-specific T cells, disrupt viral clearance, and lead to a state of fatigue for T cells [11].

Besides, Interleukin 6 (IL-6), IL-1β, and tumor necrosis factor-alpha (TNF-α), as pro-inflammatory cytokines at high concentrations, may cause shock and tissue damage in the form of cytokine storms. This phenomenon significantly contributes to the development of multiple organ dysfunction syndromes (MODS) and acute respiratory distress syndrome (ARDS) [12, 13].

The development of an antiviral mode often mediates immunity against viral infections by type-I interferons in the body. Cells detect viral attacks through intracellular and membranous pattern recognition receptors (PRR). Among these viruses, SARS-CoV-2 activates melanoma differentiation-associated protein 5 (MDA5), a retinoic acid-inducible gene I (RIG-I), and Toll-like receptors (TLRs) [14]. Oligomerization of virus-induced PRRs inactivates interferons downstream regulators and transcription factors of nuclear factor-kappa B (NF-κB), thereby producing type-I, -II, and -III IFNs [14].

The released IFN via infected cells binds to the IFN receptors of paracrine cells and alerts them to viral existence. Complexes of transcription factors are formed by activation of different members of the STAT and JAK families due to type-I and -III interferons involvement. For example, IFN-I binds to its receptor and activates JAK-1 and TYK-2, phosphorylating STAT-1 and STAT-2, respectively. Interaction of STAT-1 with STAT-2 and also IRF-9 result in the formation of the “IFN 3-stimulated gene factor (ISGF3)” transcription factor [14]. In contrast, Phosphorylated forms of STAT-1 homo-dimer, which are known as “γ-interferon activating factor” (GAF), are produced as a result of JAK1 and JAK2 activation in the IFN-II signaling pathway [15]. Transferring of GAF and ISGF3 complexes to the nucleus and subsequent rearrangement by ISG products is necessary prior to activating transcription factors [15]. Other than IFNs, STAT families have important roles in cytokine signaling pathways like IL-6 [15, 16].

Coronaviruses use a variety of mechanisms to inhibit IFN production and response [14]. SAR-CoV-2 infection is characterized by abnormal production and response to IFN-I and -III, masking IFN-related febrile symptoms and spreading the virus [17–19]. In a study conducted by Yang et al., it was found that inhibition of STAT1 phosphorylation by SARS-CoV-2 leads to impaired transcription of ISGs in dendritic cells and monocyte-derived macrophages [20].

In addition, the neutralization of type-I IFNs by autoantibodies, observed in patients with several underlying infectious diseases, predisposes them to severe viral infections. Bastard et al. identified high-titer neutralizing antibodies against IFN-ω and IFN-α2 in about 10% of COVID-19 patients with severe conditions. These antibodies were not found in infected people who were asymptomatic or had a milder phenotype or in healthy individuals [21].

Therefore, the present study was conducted to compare lymphocyte changes, the antiviral pathway of IFNs-I, and the presence of autoantibodies against IFNs-I in patients with severe and mild COVID-19 without any underlying disease to find related prognostic factors.

## Material and methods

### Study design

In this study, 100 patients with confirmed COVID-19 disease, positive real-time PCR, and chest CT SCAN who were referred to the Imam Reza hospital of Tabriz, Iran, from May until September 2021 were enlisted. Aged-match 50 healthy staff of the corona ward of the same hospital (aged 45.06 ± 10.81 years) with negative history of COVID-19 infection were also considered controls. The total number of patients participating in this experiment was divided into two groups based on mild and severe symptoms (47.64 ± 9.936 years for severe cases and 45.70 ± 10.36 years for mild cases) and the number of male participants was slightly dominant in the COVID-19 patients with severe symptoms. Patients were classified based on their mild or severe situations according to their severity of lung involvement and hospitalization time. SARS-CoV-2 patients with more than one week of hospitalization and intensive lung involvement were considered with severe symptoms. The patients were hospitalized on days 4–7 after symptoms onset. Also, the blood samples used for clinical and immunological assays were taken on the first day of hospitalization. No extra intervention was done on the patients except routine treatments used for COVID-19 patients (i.e., corticosteroids) and antiviral medications (e.g., dexamethasone and Remdesivir). Reluctance to participate in the study and any underlying diseases, such as primary immune deficiencies (PID), cancer, autoimmune diseases, and other microbial infections, were considered exclusion criteria. Designated informed written consent by the ethics Committee for emerging diseases was obtained from patients. The Ethics Committee of Tabriz University of Medical Sciences (IR.TBZMED.REC.1400.174) confirmed this study.

### Blood sampling and cell culture

About 10 ml of peripheral blood samples were collected via venipuncture from COVID-19 and healthy control subjects. Serum samples were isolated by centrifugation and stored at -80˚C for future evaluation. PBMCs were isolated from remaining heparinized blood samples using density-gradient centrifugation by 1.077 g/ml Ficoll (Histopaque; Sigma-Aldrich, Germany). It was done through 450 g centrifugation for 25 min, which was continued through twice washing by RPMI‐1640 medium (Biosera, UK). Portions of freshly-isolated PBMCs were used for immunophenotyping analysis by flow cytometry. The remained PBMCs were used for culturing in gene and protein expression assessments. PBMCs cultured in RPMI-1640 medium included heat‐inactivated fetal bovine serum (10%), streptomycin and penicillin solution (1%), phorbol myristate acetate (PMA) (10 ng/ml), and L‐glutamine (200 mM) for 48 h in the incubator (37 °C and 5% CO2). Biosafety level II plus conditions were used for all study steps, from PBMCs isolation to the following experiments.

### Flow cytometry

**Fig. 1:**
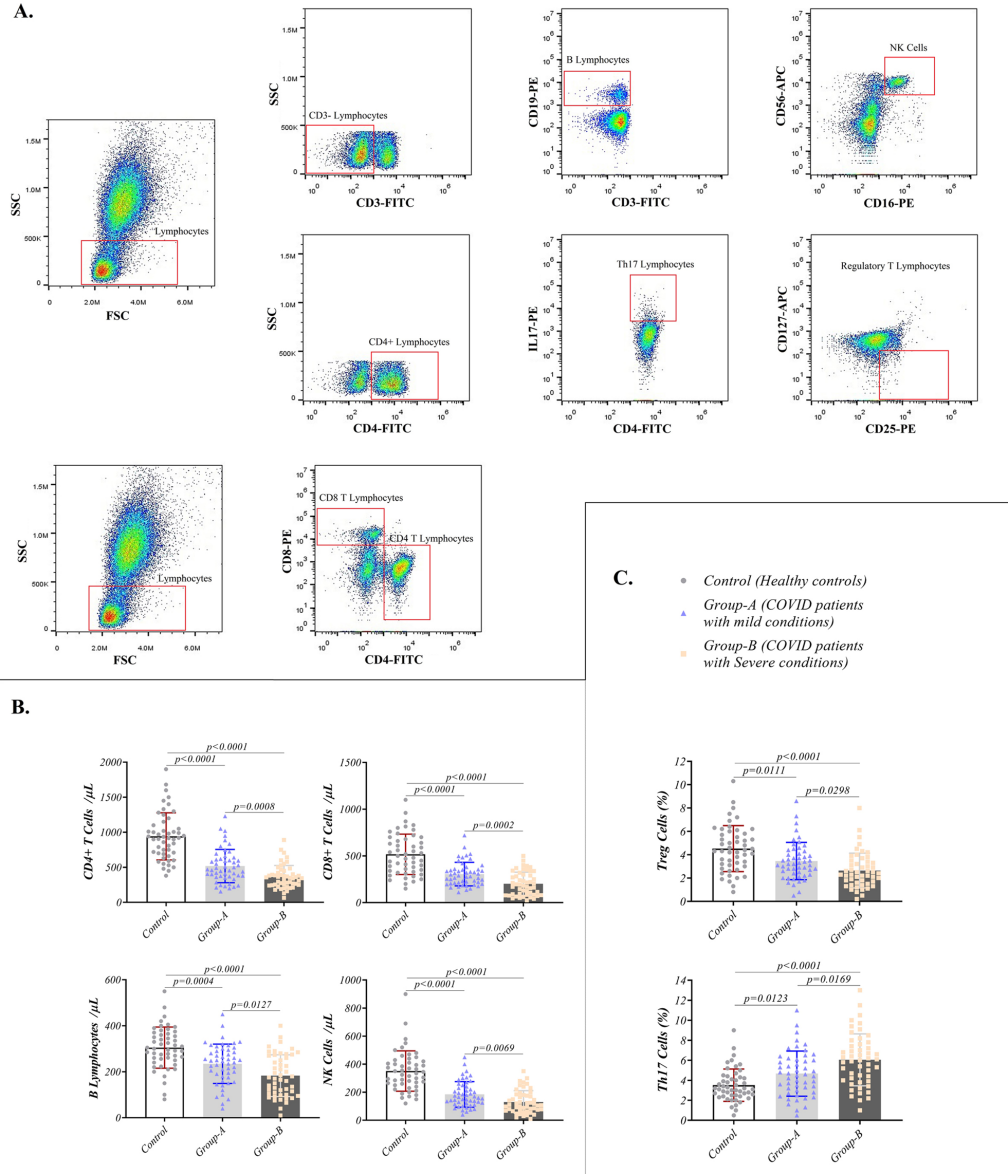
Immune-phenotyping analysis of COVID-19 patients: **A** Representative scatters show gating strategy for NK cells and different types of lymphocytes evaluation: After gating lymphocytes, CD4 + T and CD8 + T lymphocytes were analyzed based on CD4 and CD8 expression, respectively. B lymphocytes and NK cells frequency were investigated in gated CD3- lymphocytes. Th17 and Treg lymphocytes populations were assessed in CD4 + gated T lymphocytes. **B** Absolute count of NK cells, CD4 + T, CD8 + T, and B lymphocytes are presented in COVID-19 patients (mild and severe condition) and healthy controls. **C** Frequency of Th17 and Treg cells were evaluated in COVID-19 patients (mild and severe condition) and healthy controls. Graphs are presented as dot plots (three groups have the same sample size, N = 50)

Fresh collected PBMCs were used for immune-phenotyping evaluations via FACS Calibur (BD Biosciences, USA) flow cytometer between COVID-19 patients and healthy controls. CD3-FITC, CD4-FITC, CD8-PE, CD16-PE, CD19-PE, CD56-APC, CD127-APC, and il-17-PE fluorescent conjugated antibodies were utilized to evaluate different subsets of T lymphocytes (e.g., CD8 + T lymphocytes, CD4 + T lymphocytes, Th17 lymphocytes, and Treg lymphocytes), B lymphocytes, and NK cells frequencies. Frequencies of CD4 + T and CD8 + T lymphocytes were assessed by analyzing the gated lymphocytes based on their CD4 and CD8 expression. CD4 + CD8- lymphocytes were considered CD4 + T lymphocytes and CD4- CD8 + lymphocytes as CD8 + T lymphocytes. CD3 + CD19 + B lymphocytes and CD16 + CD56 + NK cells were evaluated based on CD19 expression for B lymphocytes and CD16 and CD56 expression for NK cells on gated CD3 + lymphocytes. In addition, CD4 + IL-17 + Th17 lymphocytes and CD4 + CD25 + CD127- Treg lymphocytes were studied based on IL-17 expression for Th17 lymphocytes and CD25 positive and CD127 negative expression for Treg lymphocytes on CD4 + gated lymphocytes. Gating strategies of all cell subsets are presented in Fig. 1a. Required isotype control antibodies were utilized to validate gating for different cell types. All antibodies were purchased from the eBioscience company (San Diego, USA).

### RNA extraction and real-time PCR

RNeasy Kits (Qiagen, UK) were used to extract PBMC’s total RNA. Complementary DNA (cDNA) was synthesized using extracted RNA and QuantiTect reverse transcription kit (Qiagen, UK), followed by evaluating RNA quality and quantity via 260/230 and 260/280 absorbance ratio by nanodrop spectrophotometer (Agilent Technologies, CA). Synthesized cDNAs were used to evaluate the expression level of *IFN-α*, *IFN-β*, *JAK-1*, *TYK-2*, *STAT-1*, *STAT-2*, *IRF-3*, and *IRF-7* genes’ expression level studied groups.

A high-performance real-time PCR LightCycler® System (Roche, Germany) was applied to evaluate desired genes expression using the SYBER Green protocol. β-Actin was considered as normalizing internal control. The expression level of targeted genes was calculated based on 2^−△△CT^ formula using threshold cycle (Ct) values. Finally, the amplification validity was verified by electrophoresis of PCR products on 2% agarose gel.

### Protein extraction and western blotting

Western blotting analysis was performed to evaluate STAT-1, pSTAT-1, STAT-2, pSTAT-2, JAK-1, pJAK-1, TYK-2, pTYK-2, IRF-3, and IRF-7 expressions at the protein level. For this purpose, first, PBMCs proteins were extracted using RIPA lysis buffer (Santa Cruz, the USA) according to the manufacturer’s instructions. After protein lysate preparation, SDS–polyacrylamide gel was electrophoresed on protein lysate products, followed by transferring the proteins on a polyvinylidene fluoride (PVDF) membrane using a wet transfer system (BioRad, USA). Transferred PVDF papers were used to measure desired proteins expression through the western blot technique. In this technique, the transferred PVDF paper is blocked by 3% bovine serum albumin (BSA) containing TBS-Tween 20% blocking buffer for 2 h at room temperature. Then, washing, primary antibody incubation (overnight at 4), washing, secondary horseradish conjugated antibody incubation (1 h at room temperature), and final washing steps are performed, in the order of their appearance. Afterward, an electrochemiluminescence (ECL) kit was used to enhance the desired protein bands, and an image capturing system (SabzCo, Iran) was used to visualize these bands. Eventually, the ImageJ software ver.1.3 (National Institutes of Health) was used to analyze the protein bands’ intensity. All antibodies and ECL kits were purchased from Abcam Company (Abcam, USA) and utilized according to the manufacturer’s guidance.

### Enzyme-linked immunosorbent assay (ELISA)

Secreting levels of IFN-α, IFN-β, IL-1b, IL-6, TNF-α, IL-2R, IL-17, IL-10 cytokine, and anti-IFN-α autoantibody in extracted serum samples were investigated by related ELISA kits. These kits are MyBioSource (San Jose, CA, USA) for cytokines and BioVision (San Francisco, CA, USA) for anti-IFN-α autoantibody. According to the manufacturer’s procedures, 450 nm wavelength was applied to read the absorbance of samples using an iMark microplate absorbance reader (Bio-Rad, USA), followed by using a calibrated standard curve to calculate concentrations.

### Statistical analysis

Qualitative and quantitative data were compared between three groups using the Chi-square, Brown-Forsythe, and welch ANOVA Dunnett’s T3. All data were expressed as median (Min–Max) or mean (± SD) based on data type, and p < 0.05 was considered the significant level in the studied groups. Version 8.0 of GraphPad Prism software was used to analyze and illustrate statistical graphs (GraphPad, USA).

## Results

### Demographical and laboratory findings of studied populations

In addition to 100 (50 severe and 50 mild) COVID-19 patients, 50 healthy controls were included with matched ages in this study. According to the demographic table (Table 1), significant differences are observed in body mass index (BMI), systolic and diastolic blood pressure (SBP and DBP), high-density lipoprotein (HDL), creatinine, and glomerular filtration rate (GFR) in three studied groups. COVID-19 patients had higher fasting blood sugar (FBS) (*p* < 0.0001 for severe and *p* = 0.0356 for mild symptoms groups), triglyceride (TG) (*p* < 0.0001 for severe and *p*  = 0.027 for mild symptoms groups), cholesterol (*p* = 0.0003 for severe and *p* = 0.0227 for mild symptoms groups), and low-density protein (LDL) (*p* = 0.0146 for severe and *p* = 0.0005 for mild symptoms groups) levels compared to healthy controls. Also, COVID-19 patients with severe symptoms had higher FBS and TG levels than patients with mild conditions (*p* = 0.0383 and *p* = 0397, respectively). Albumin concentration analysis showed a significant decrease in COVID-19 patients with severe conditions compared to the control group (*p* = 0.0004). Studying the complete blood count (CBC) results showed a desirable reduction in white blood cells (WBC) (*p*  = 0.0038 for severe and *p* = 0.0017 for mild symptoms groups) and lymphocytes (*p* < 0.0001 for severe and *p* < 0.0001 for mild symptoms groups) in COVID-19 patients compared to healthy individuals. Also, the lymphocyte count in COVID-19 patients with severe symptoms was lower than in patients with mild conditions (*p* < 0.0001). However, there were no significant changes in neutrophil count between the three groups. Details of demographic and laboratory findings are presented in Table 1.

**Table 1:** Demographics of COVID-19 patients and healthy controls

Parameter	G1 mean ± SD (N = 50)	G2 mean ± SD (N = 50)	G3 mean ± SD (N = 50)	Significance Level		
				G1 vs. G2	G1 vs. G3	G2 vs. G3
Age (Min–Max) (Male–Female)	45.06 ± 10.81 (28–66) (23–27)	45.70 ± 10.36 (25–64) (25–25)	47.64 ± 9.936 (30–67) (29–21)	NS	NS	NS
WBC (count/μl) (Min–Max)	6314 ± 1577 (3200–9850)	5295 ± 1261 (2890–8200)	5373 ± 1227 (3040–8050)	0.0017	0.0038	NS
Neutrophil (count/μl) (Min–Max)	4326 ± 1128 (1520–6900)	3914 ± 1092 (1730–6460)	4209 ± 1099 (1620–6610)	NS	NS	NS
Lymphocyte (count/μl) (Min–Max)	1639 ± 529.4 (770–3220)	1000 ± 325.3 (300–1865)	704.4 ± 268.8 (110–1500)	< 0.0001	< 0.0001	< 0.0001
BMI (kg/m2)	25.48 ± 2.144	25.40 ± 1.801	26.03 ± 2.008	NS	NS	NS
SBP (mmHg)	114.2 ± 11.39	115.8 ± 11.52	116.7 ± 13.01	NS	NS	NS
DBP (mmHg)	73.76 ± 6.989	75.10 ± 7.424	74.26 ± 7.695	NS	NS	NS
FBS (mg/dl)	99.80 ± 16.05	111.6 ± 28.15	128.7 ± 38.65	0.0356	< 0.0001	0.0383
TG (mg/dl)	130.1 ± 40.09	154.8 ± 51.64	178.0 ± 39.81	0.0270	< 0.0001	0.0397
Cholesterol (mg/dl)	156.5 ± 39.39	177.5 ± 37.69	185.6 ± 31.90	0.0227	0.0003	NS
HDL (mg/dl)	51.04 ± 6.163	49.56 ± 8.501	48.03 ± 7.241	NS	NS	NS
LDL (mg/dl)	98.76 ± 29.62	120.2 ± 24.56	117.7 ± 35.96	0.0005	0.0146	NS
Albumin (g/dl)	3.510 ± 0.2190	3.370 ± 0.4063	3.298 ± 0.3034	NS	0.0004	NS
Creatinine (mg/dl)	1.619 ± 1.061	1.441 ± 0.8133	1.406 ± 0.6645	NS	NS	NS
GFR (cc/min)	74.48 ± 21.66	71.52 ± 27.78	65.52 ± 22.51	NS	NS	NS
Clinically COVID-19 Positive Subjects (CT-Scan/PCR)	- -	50 (16–50)	50 (50–50)	- -	- -	- -

Data are presented as mean ± SD

*p* < 0.05 was considered as statistically significant

G1: Healthy Controls; G2: Patients with mild symptoms; G3: Patients with severe symptoms; WBC: White blood cell; BMI: Body mass index; SBP: Systolic blood pressure; DBP: Diastolic blood pressure; FBS: Fasting blood sugar; TG: Triglyceride; HDL: High density lipoprotein; LDL: Low density lipoprotein; GFR: Glomerular filtration rate

### The severity of the symptoms in patients in association with the lymphocytes reduction

Immune-phenotyping results of COVID-19 patients showed an obvious decrease in different types of lymphocytes in patients with severe symptoms. Analysis of CD4 + T lymphocytes, CD8 + T lymphocytes, and NK cells revealed a significant decrease in the absolute count of these cells in COVID-19 patients compared to healthy controls (*p* < 0.0001 for all described groups). In addition, COVID-19 patients with severe conditions had lower counts of these cells than patients with mild conditions (*p* = 0.0008 for CD4 + T lymphocytes, *p* = 0.0002 for CD8 + T lymphocytes, and *p* = 0.0069 for NK cells) (Figs. 1b and Table 2). However, there were no remarkable changes in these lymphocytes percent between the three groups. Along with T lymphocytes data, a significant reduction was observed in B lymphocytes absolute count (*p* < 0.0001 for severe and *p* = 0.0004 for mild symptoms groups) and percentage (*p* < 0.0001 for severe and *p* < 0.0001 for mild symptoms groups) in patients groups compared with healthy individuals (Fig. 1b and Table 2). B lymphocytes had a reduced number in patients with severe conditions than in those with mild symptoms (*p* = 0.0127).

**Table 2:** Immunological features of COVID-19 patients and healthy controls

Parameter	G1 (N = 50) Median (Min–Max)	G2 (N = 50) Median (Min–Max)	G3 (N = 50) Median (Min–Max)	Significance Level		
				G1 vs. G2	G1 vs. G3	G2 vs. G3
*Flowcytometry*						
CD4 + T Lymphocytes (count/μl)	917.5 (380–1900)	465 (155–1230)	347.5 (65–890)	< 0.0001	< 0.0001	0.0008
CD8 + T Lymphocytes (count/μl)	490 (150–1100)	285 (110–720)	186.0 (25–500)	< 0.0001	< 0.0001	0.0002
B Lymphocytes (count/μl)	302.5 (80–550)	246.5 (40–450)	172.5 (10–400)	0.0004	< 0.0001	0.0127
NK Cells (count/μl)	340 (120–900)	162.5 (40–450)	109 (10–350)	< 0.0001	< 0.0001	0.0069
	G1 mean ± SD (N = 50)	G2 mean ± SD (N = 50)	G3 mean ± SD (N = 50)			
CD4 + T Lymphocytes (%)	42.54 ± 7.308	40.93 ± 7.131	39.31 ± 9.149	NS	NS	NS
CD8 + T Lymphocytes (%)	24.71 ± 7.741	22.70 ± 7.914	23.18 ± 8.236	NS	NS	NS
B Lymphocytes (%)	12.23 ± 2.406	9.694 ± 2.340	9.136 ± 3.201	< 0.0001	< 0.0001	NS
NK Cells (%)	15.17 ± 7.504	12.81 ± 8.205	12.87 ± 7.538	NS	NS	NS
Treg Cells (%)	4.524 ± 1.967	3.458 ± 1.595	2.648 ± 1.489	0.0111	< 0.0001	0.0298
Th17 Cells (%)	3.512 ± 1.621	4.668 ± 2.254	6.044 ± 2.598	0.0123	< 0.0001	0.0169
*Relative Gene Expression (Fold Change)*						
*IFN-α*	1.000 ± 0.09112	0.8592 ± 0.3361	0.5844 ± 0.3282	0.0177	< 0.0001	0.0002
*IFN-β*	1.000 ± 0.1020	0.8094 ± 0.4074	0.6324 ± 0.2801	0.0066	< 0.0001	0.0388
*JAK-1*	1.000 ± 0.08152	0.9100 ± 0.2939	0.7220 ± 0.4729	NS	0.0004	NS
*TYK-2*	1.000 ± 0.07461	0.8752 ± 0.2590	0.5518 ± 0.4402	0.0054	< 0.0001	< 0.0001
*STAT-1*	1.000 ± 0.07835	0.8892 ± 0.3473	0.6892 ± 0.5016	NS	0.0002	NS
*STAT-2*	1.000 ± 0.05379	0.7664 ± 0.2876	0.5234 ± 0.3018	< 0.0001	< 0.0001	0.0002
*IRF-3*	1.000 ± 0.06862	0.7036 ± 0.3234	0.5492 ± 0.2803	< 0.0001	< 0.0001	0.0364
*IRF-7*	1.000 ± 0.1209	0.7684 ± 0.4125	0.5644 ± 0.3296	0.0010	< 0.0001	0.0224
*Western Blotting (%)*						
STAT-1	63.56 ± 23.78	57.96 ± 33.92	43.76 ± 26.14	NS	0.0004	NS
STAT-2	74.64 ± 17.53	54.76 ± 22.68	40.94 ± 31.20	< 0.0001	< 0.0001	0.0385
JAK-1	65.82 ± 21.38	59.74 ± 32.22	44.46 ± 15.64	NS	< 0.0001	0.0106
TYK-2	69.68 ± 17.56	60.14 ± 37.22	41.92 ± 21.65	NS	< 0.0001	0.0110
pSTAT-1	62.14 ± 21.46	47.96 ± 23.02	37.54 ± 17.56	0.0058	< 0.0001	0.0372
pSTAT-2	66.24 ± 16.49	52.2 ± 21	39.44 ± 21.34	0.0010	< 0.0001	0.0098
pJAK-1	62.42 ± 19.25	50.18 ± 21.96	40.1 ± 15.59	0.0114	< 0.0001	0.0285
pTYK-2	63.14 ± 17.05	52.3 ± 21.58	38.74 ± 19.46	0.0192	< 0.0001	0.0041
IRF-3	70.20 ± 26.63	53.74 ± 22.76	36.62 ± 22.01	0.0038	< 0.0001	0.0007
IRF-7	73.56 ± 30.15	54.72 ± 33.04	30.66 ± 14.75	0.0109	< 0.0001	< 0.0001
*Enzyme-Linked Immunosurbent Assay (ELISA)*						
IFN-α (pg/ml)	26.63 ± 19.02	15.45 ± 13.92	11.13 ± 9.518	0.0035	< 0.0001	NS
IFN-β (pg/ml)	6.116 ± 4.259	3.194 ± 1.492	2.582 ± 1.243	< 0.0001	< 0.0001	NS
IL-1β (pg/ml)	7.984 ± 4.239	12.63 ± 7.090	20.20 ± 13.94	0.0005	< 0.0001	0.0031
IL-6 (pg/ml)	7.467 ± 2.995	13.26 ± 9.594	33.07 ± 37.88	0.0004	< 0.0001	0.0021
TNF-α (pg/ml)	3.682 ± 1.549	10.99 ± 13.45	27.90 ± 29.71	0.0011	< 0.0001	0.0015
IL-2R (U/ml)	467.2 ± 200.9	618.5 ± 212.8	943.2 ± 326.0	0.0012	< 0.0001	< 0.0001
IL-17 (pg/ml)	11.55 ± 5.716	16.34 ± 7.485	21.28 ± 9.411	0.0016	< 0.0001	0.0137
IL-10 (pg/ml)	5.482 ± 1.556	5.624 ± 3.142	10.41 ± 7.814	NS	0.0002	0.0005
IFN-α auto-antibody positive cases	0	2	14	NS	< 0.0001	0.001

Data are presented as mean ± SD

*p* < 0.05 was considered as statistically significant

G1: Healthy Controls; G2: Patients with mild symptoms; G3: Patients with severe symptoms; NK Cells: Natural killer cells; Treg Cells: Regulatory T lymphocytes; Th17 Cells: T helper 17 lymphocytes; IFNs: interferons; JAK-1: Janus kinase 1; TYK-2: Tyrosine kinase 2; STATs: Signal transducer and activator of transcriptions; IRFs: The transcription factor interferon regulatory factors; ILs: Interleukins; TNF-α: Tumor necrosis factor alpha; IL-2R: Interleukin 2 receptor

The frequency of Treg lymphocytes showed a remarkable decrease in these patients compared to healthy controls (*p* < 0.0001 for severe and *p* = 0.0111 for mild symptoms groups) and also in patients with severe symptoms (*p* = 0.0298) compared to those with mild symptoms patients (Fig. 1c and Table 2). Unlike Treg lymphocytes, Th17 lymphocytes demonstrated a higher frequency in patients toward controls (*p* < 0.0001 for severe and *p* = 0.0123 for mild symptoms groups) and in patients with severe symptoms (*p* = 0.0169) compared to those with mild symptoms patients (Fig. 1c and Table 2).

### Reducing IFN-I signaling pathway in COVID-19 patients

**Fig. 2:**
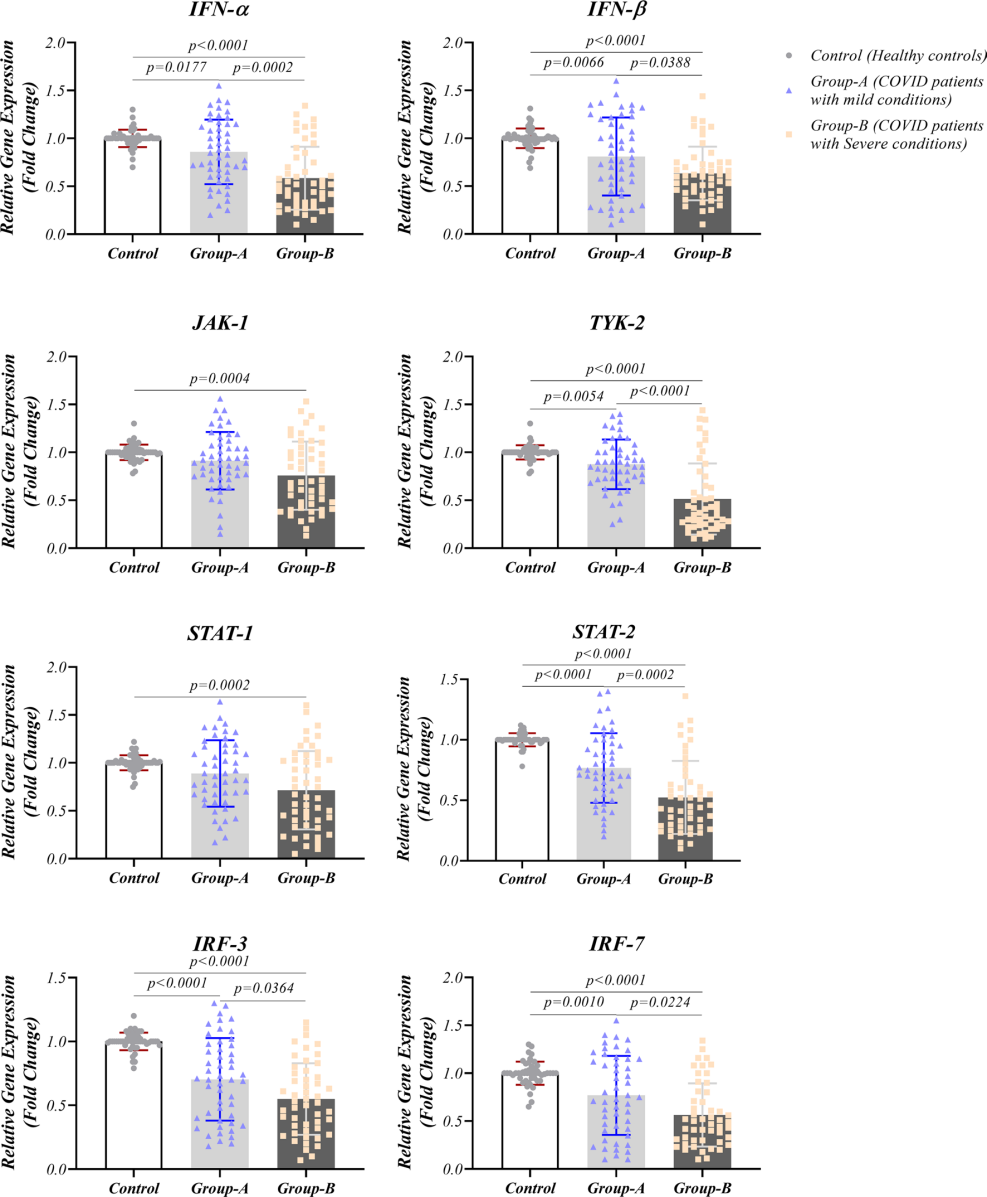
Gene expression pattern of antiviral molecules in COVID-19 patients: Expression levels of *IFN-α, IFN-β, JAK-1, TYK-2, STAT-1, STAT-2, IRF-3*, and *IRF-7* as antiviral elements are evaluated at mRNA status in COVID-19 patients (mild and severe condition) and healthy controls via real-time PCR. Graphs are presented as dot plots (three groups have the same sample size, N = 50)

**Fig. 3:**
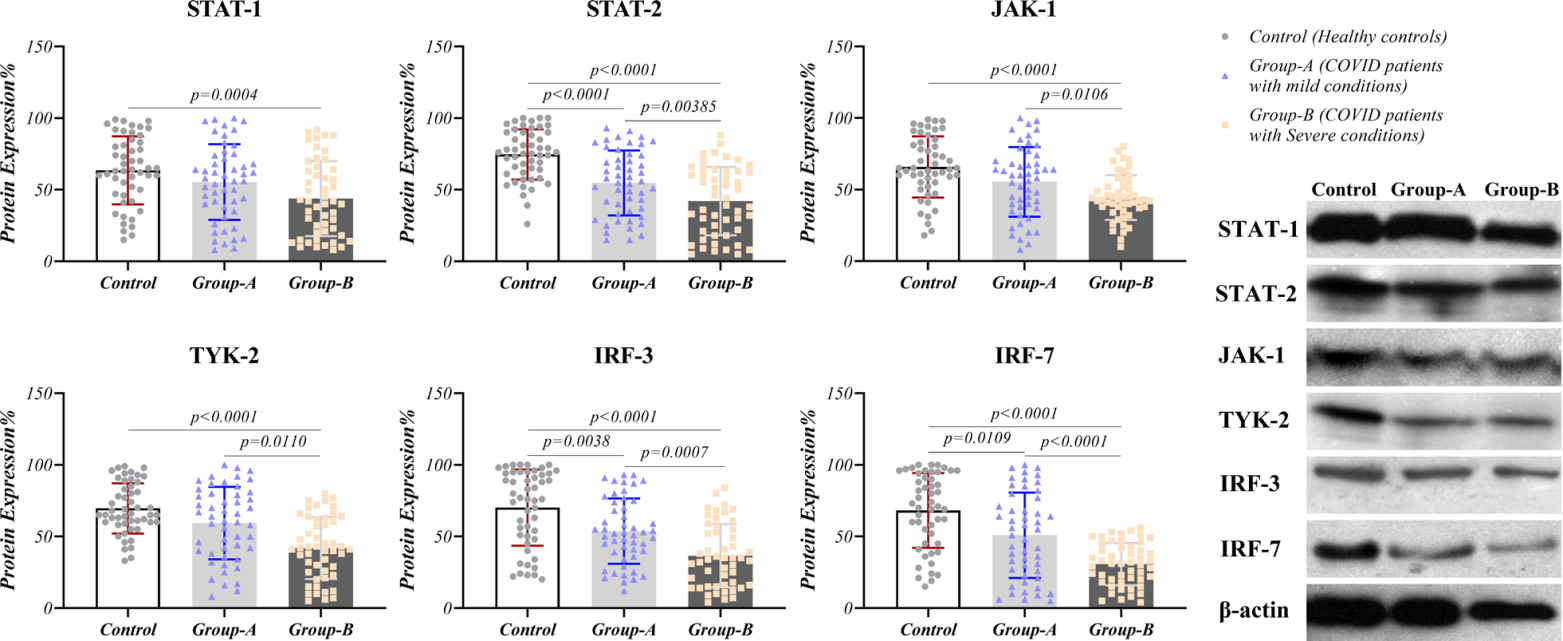
Protein expression pattern of antiviral molecules in COVID-19 patients: Expression levels of JAK-1, TYK-2, STAT-1, STAT-2, IRF-3, and IRF-7 as antiviral elements are evaluated at protein status in COVID-19 patients (mild and severe condition) and healthy controls using the western blotting technique. Graphs are presented as dot plots (three groups have the same sample size, N = 50)

**Fig. 4:**
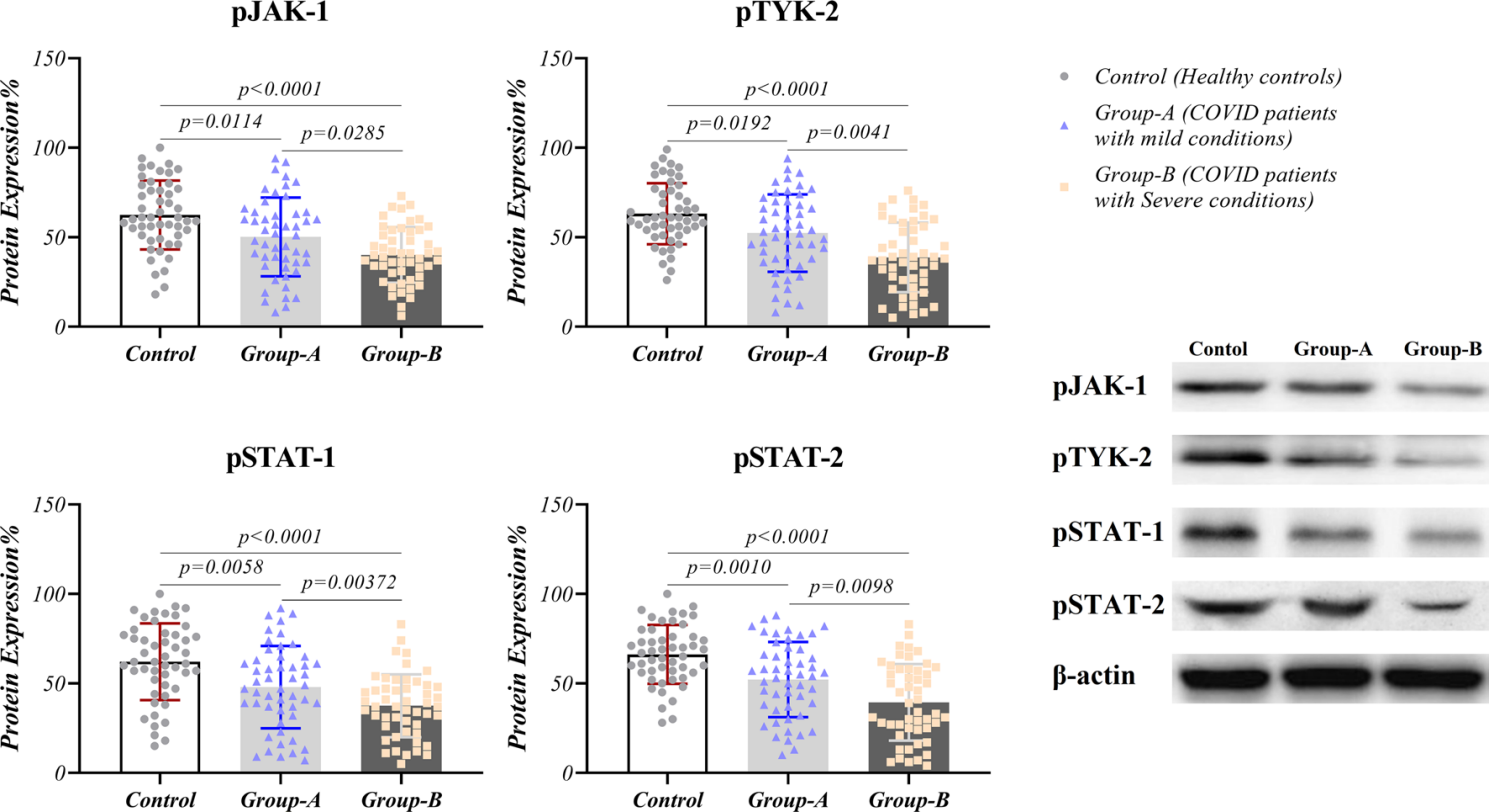
Expression pattern of phosphorylated proteins in COVID-19 patients: Expression levels of pJAK-1, pTYK-2, pSTAT-1, and pSTAT-2 were evaluated at protein status in COVID-19 patients (mild and severe condition) and healthy controls using the western blotting technique. Graphs are presented as dot plots (three groups have the same sample size, N = 50)

IFN-I signaling pathway of COVID-19 patients was investigated in both RNA and protein levels using real-time PCR and western blotting techniques, respectively. The mRNA expression analysis of IFN-α (*p* < 0.0001 for severe and *p* = 0.0177 for mild symptoms groups) and IFN-β (*p* < 0.0001 for severe and *p* = 0.0066 for mild symptoms groups) demonstrated a significant decrease in the patients compared to the healthy population. Additionally, patients with severe conditions had lower expression levels of these genes compared to patients with conditions (Fig. 2 and Table 2). JAK-1 had reduced expression in severe-condition patients compared with the healthy group in both mRNA (*p* = 0.0004) and protein (*p* < 0.001) levels and compared with mild-condition patients (*p* = 0.0106) only at protein state (Figs. 2 and 3 and Table 2). TYK-2 expression revealed reduced status in severe condition patients compared to the healthy group and patients with mild symptoms at both mRNA (*p* < 0.0001 for both groups) and protein (p < 0.0001 for control and *p* = 0.011 for mild-symptoms patients) levels (Figs. 2 and 3). COVID-19 patients with severe symptoms showed a lower expression pattern of STAT-1 compared to the healthy group at mRNA (*p* = 0.0002) and protein (*p* = 0.0002) status (Figs. 2 and 3). Our results showed decreased expression of STAT-2, IRF-3, and IRF-7 genes and proteins in the patients’ group compared to healthy controls. In addition, these products had a lower state in severe condition patients compared to the other patient group. The significance level for STAT-1, IRF-3, and IRF-7 at mRNA level between severe and mild conditions patients were *p* = 0.0002, *p* = 0.0364, and *p* = 0.0224, respectively. Also, these values were *p* = 0.0385, *p* = 0.0007, and *p* < 0.0001 at protein level between patients group. Phosphorylated forms of JAK-1 and TYK-2 had reduced level in patients with the severe condition compared to mild-condition patients (*p* = 0.0285, and *p* = 0.0041, for pJAK-1 and pTYK-2, respectively) and healthy controls (*p* < 0.0001 for both proteins). In addition, phosphorylated forms of STAT-1 and STAT-2 had reduced levels in patients with the severe conditions compared to mild-condition patients (*p* = 0.0372, and *p* = 0.0098, for pSTAT-1 and pSTAT-2, respectively) and healthy controls (*p* < 0.0001 for both proteins). The details of gene and protein expressions are provided in Figs. 2, 3, and 4 and Table 2.

### Serum cytokines pattern showed a clue of hyper-inflammation in COVID-19 patients

**Fig. 5:**
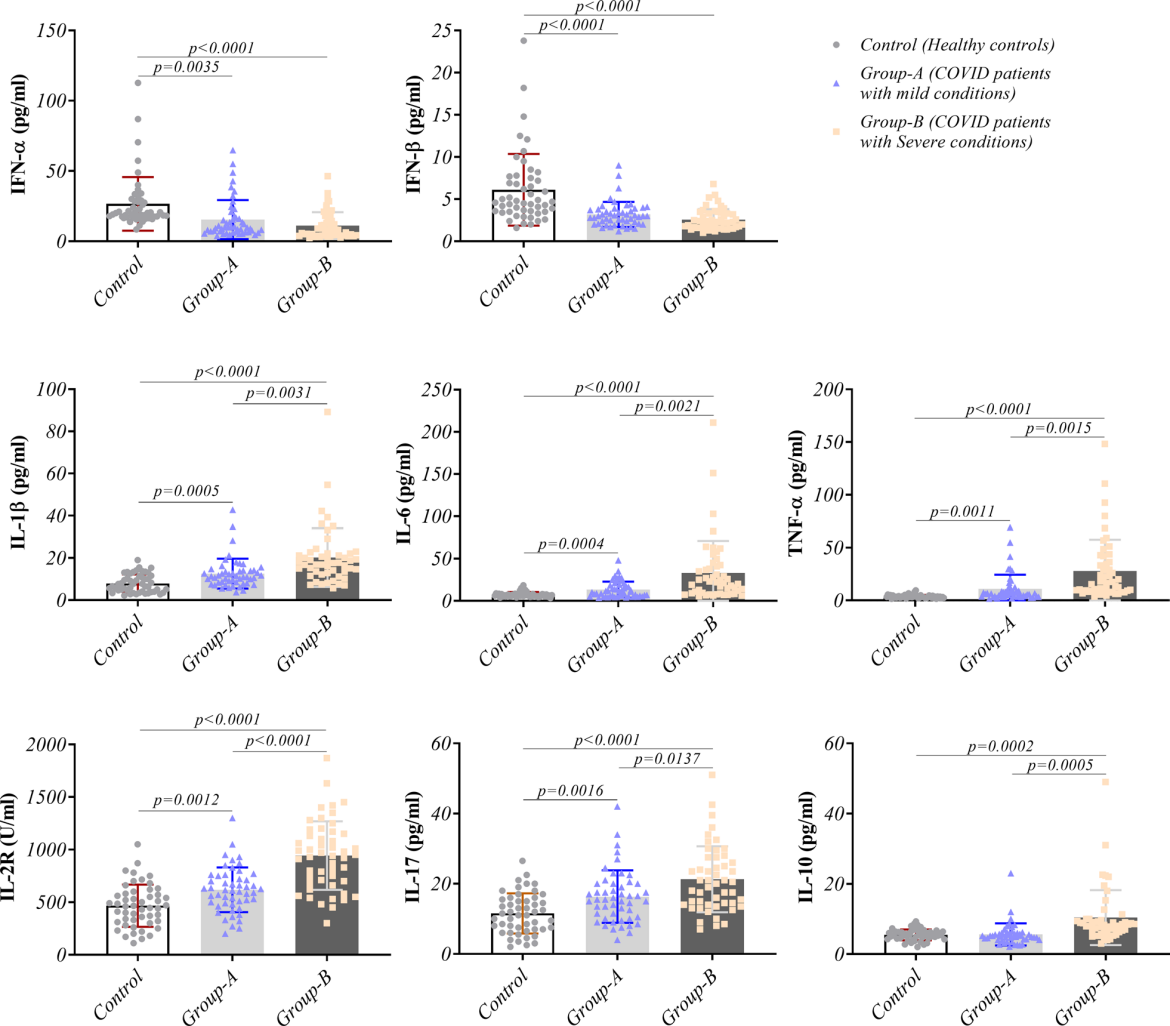
Evaluation of secreted cytokines level in COVID-19 patients: The concentration of IFN-α, IFN-β, IL-1β, IL-6, TNF-α, IL-2R, IL-17, and IL-10 cytokines were assessed in COVID-19 patients (mild and severe condition) and healthy controls by ELISA. Graphs are presented as dot plots (three groups have the same sample size, N = 50)

The ELISA technique was applied to evaluate the concentration of serum-secreted cytokines. The results showed a significant decrease in IFN-α (*p* < 0.0001 for severe and *p* = 0.0035 for mild symptoms group) and IFN-β (*p* < 0.0001 for both patient groups) concentrations in the patient groups compared to the controls. There was no remarkable change between the patient groups for these cytokines. IL-1β, IL-6, TNF-α, IL-2R, and IL-17 as inflammatory cytokines had a remarkably increased concentration in the sera of patient groups compared to healthy ones. Notably, the concentration of these cytokines increased even in patients with severe conditions compared to patients with mild symptoms (Fig. 4 and Table 2). Our investigation showed an increased concentration of IL-10 in COVID-19 patients with severe conditions compared to the healthy group (*p* = 0.0002) and patients with mild symptoms (*p* = 0.0005). The details of cytokines concentration are provided in Fig. 5 and Table 2.

### Existence of anti-IFN-α autoantibody in severe condition COVID-19 patients

**Fig. 6:**
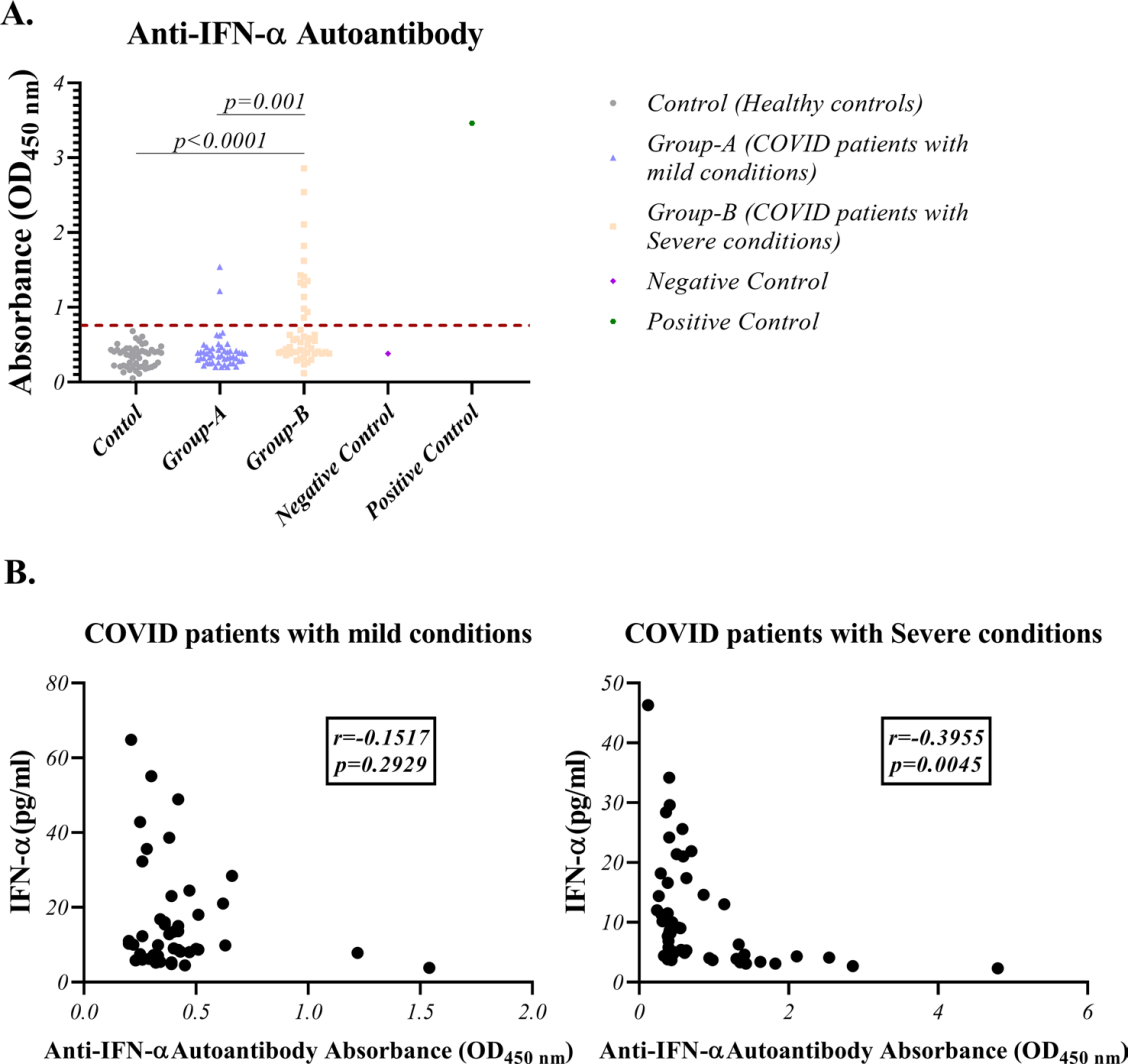
Investigation of anti-IFN-α autoantibody existence in COVID-19 patients and its correlation with IFN-α level: **A** Evaluation of anti-IFN-α autoantibody existence in COVID-19 patients (mild and severe condition) and healthy controls using ELISA (Median values for health controls, COVID-19 patients with mild and severe conditions are 0.375, 0.36, and 0.5150, respectively); Graphs are presented as dot plots with negative and positive controls. The calculated cut-off index was 0.748 nm and presented by a dotted line. Three groups have the same sample size (N = 50). **B** Correlation of IFN-α serum level and anti-IFN-α autoantibody existence in COVID-19 patients with severe and mild symptoms using Pearson r correlation assay

Evaluation of the studied groups’ serums to find anti-IFN-α autoantibody revealed that 14 cases of COVID-19 patients with severe symptoms had this type of antibody. This result for patients with mild symptoms was 2 cases (*p* = 0.001) and was negative for the healthy control group (*p* < 0.0001) (Fig. 6a and Table 2). Also, the correlation study showed a significant reverse correlation between the presence of anti-IFN-α autoantibodies and the levels of IFN-α in COVID-19 patients with severe conditions (p = 0.0045) (Fig. 6b).

## Discussion

The COVID-19 pandemic has become the world’s main life-threatening challenge in the third decade of the twenty-first century. More than five million people have died around the globe due to SARS-CoV-2 infection until now. Despite numerous studies on the molecular structure of the virus, the pathogenesis of the disease, possible therapies, and the development of high-performance vaccines, the disease has not yet been eradicated, and we witness the daily death of thousands of people around the world.

Like any other viral infection, the innate and acquired immune system and its antiviral activity are key factors in combating this infection [22]. CD4 + helper T cells improve immune response against viral infection by secreting immune-stimulatory cytokines to elevate CD8 + T lymphocytes’ cytotoxic activity, and B cells specific neutralizing antibody secretion [8, 10]. However, in some of these viral infections (e.g., SARS-CoV-2), we see a decrease in lymphocytes count, dysregulation of immune system balance, and a decrease in antiviral responses due to genetic factors and the virus’ ability to escape the immune system in some populations [11, 21, 23]. Despite numerous previous studies on COVID-19 infection in different populations, the exact reason for severe cytokine response and the lymphocytes reduction in these patients (especially in patients with severe symptoms and no underlying diseases) has not been understood yet. To find more about these issues, we investigated the immune profile and IFN-I signaling pathway of COVID-19 patients with severe conditions with no history of underlying diseases.

The results showed a desirable reduction in all types of lymphocytes, including NK cells, B lymphocytes, CD8 + T lymphocytes, and CD4 + T lymphocytes in COVID-19 patients, especially those with severe symptoms. Viral-related T cell reduction in some virus infections (e.g., Measles) is due to the production of T cell proliferation-blocking proteins. These proteins are encoded by the virus genome and the cytotoxic effect of the virus on these cells [24]. Several studies have reported a decrease in the number of lymphocytes subtypes with normal or elevated neutrophil counts in COVID-19 patients [25–28]. For instance, in one of the first studies on COVID-19 patients, Qin et al. [25] showed that SARS-CoV2 targets T lymphocytes. They also showed that surveillance of neutrophil–lymphocyte ratio and subtypes of lymphocytes might be helpful in the early detection of severe patients, accurate diagnosis, and better treatment of these patients. In a study conducted by Liu et al. [28], a high degree of lymphopenia and intense secretion of pro-inflammatory cytokines were observed in severe COVID-19 patients rather than mild cases. Besides, they associated these results with disease severity. In addition to lymphopenia, we observed a high frequency of Th17 cells with a lower percentage of Treg cells in our patient groups. This finding is in line with the results of Tahmasebi et al. [29, 30]. SARS-CoV-2 relating lymphopenia probably occurs by virus-encoded genome products that are not well understood. They may also be attributed to hyper-activating lymphocytes followed by subsequent exhaustion and reduction of these cells [31]. Overall, more targeted studies are required in this regard.

Furthermore, we found more reduced secretion and expression of IFN-α, IFN-β cytokines, and IFN-I related signaling pathway molecules in COVID-19 patients with severe conditions than those with mild conditions and controls, respectively. Following viral infection, type-I IFNs, including IFN-α, -β, and -ω, are quickly induced and orchestrated by an antiviral response via the JAK-STAT signaling pathway and expression of ISGs [32]. Moreover, impaired production of IFN-a had been observed in most severe COVID-19 patients. This type of IFN-Is reduction resembles respiratory syncytial virus infection observed in young children with severe symptoms [33]. However, it is different from the response triggered by other types of respiratory-involved viruses like influenza A or human Parainfluenza viruses, which are determined by a robust IFN-Is response in vitro studies [19]. Some studies on IFN-I responses to SARS-CoV infections show a reduction in IFN-I secretion and its signaling molecules [19, 34–37]. For example, Blanco-Melo et al. [19] showed that lower IFN-I, IFN-III, and ISG responses and induction in the production of pro-inflammatory cytokines in SARS-CoV-2 infected different cells types. Reported data from animal models and cellular studies indicated that SARS-CoV-2 could inhibit the induction of IFN-Is and IFN-III [19]. These data suggest establishing effective mechanisms by SARS-CoV-2 to stop host IFN production. In another study, Frieman et al. [36] indicated the inhibitory role of SARS-CoV ORF6 in blocking STAT-1 and IFN-I signaling pathways in an in-vitro assay. SARS-CoV-2 proteins are similar to SARS-CoV-1 ones structurally and, therefore, may have the same effects on the production and responses of IFNs. Nevertheless, there is an important difference in this regard: the premature stop codon in the ORF3b gene of SARS-CoV-2 results in a truncated protein (22aa) compared with the SARS-CoV-1 ORF3b (154aa) protein. This issue may more severely reduce signaling and subsequently response of IFN-I [34].

The other part of our study is about screening the pro-inflammatory cytokines value in these patients. The results showed an elevated concentration of IL-1β, IL-6, TNF-α, IL-2R, IL-17, and IL-10 cytokines in the serum of COVID-19 patients. Except for IL-10, the others are pro-inflammatory cytokines, and their elevation in these patients has been reported in several studies [28, 38, 39]. Increasing the IL-10 levels as an immune-suppressor cytokine in these patients seems somehow strange because of the hyper-inflammatory profiles of these patients. In this respect, two points may help us to consider this elevation as a result of SARS-CoV-2 infection. The role of IL-10 in promoting Th2 response to antibody production occurring in viral infections is the first point [40]. The second reason might be the immune system attempts to save the host by reducing hazardous hyperinflammation. Overall, further investigations are needed to find more reliable information about this contrast. In a study of COVID-19 patients by Mehta et al. [41], it was observed that a hyperactivated immune system with a hazardous elevation in inflammatory cytokines like IL-1b, IL-2R, IL-6, and TNF-α had been developed in SARS-CoV-2 patients with severe conditions. Organ dysfunction that occurs due to COVID-19 infection is not the result of cytokine storm loneliness because of the inadequate elevated inflammatory response needed to combat SARS-CoV-2 infection. Leisman et al. reported this finding in a meta-analysis study [42].

As the final step of this research, we investigated the participants’ sera to find possible anti-IFN-α antibodies. Our results showed that 28% of patients with severe conditions had this type of autoantibodies and a negative correlation was observed between autoantibodies production and IFN-α serum level in this group. Bastard et al. [21] reported for the first time that 10.2% of critically COVID-19 patients had anti-IFN-I autoantibodies. These autoantibodies had been reported in autoimmune like systemic lupus erythematosus (SLE) and behçet diseases [43, 44]. These autoantibodies also are produced in a very low minority of healthy individuals. In a recent study, Goncalves et al. [45] showed that 18% of critically COVID-19 patients had these autoantibodies in their sera. They also attributed the presence of these autoantibodies to a superior danger of developing a severe form of COVID-19.

Some hypotheses are proposed to demonstrate variations in IFN-I responses to infection. Certain diseases are risk factors for developing a severe form of COVID-19 that could negatively affect the production of IFN and exacerbate inflammation [46, 47]. Genetic susceptibilities like monogenic disorders in children and adults can be suspected to involve impaired IFN-I signaling pathways associated with a life-threatening viral infection such as influenza [48, 49]. Production of neutralizing anti-IFN-I autoantibodies in COVID-19 patients with and without underlying diseases is another risk factor associated with low IFN-Is levels and disease severity. Moreover, recognizing patients with insufficient IFN-Is response due to impaired IFN-Is induction and/or neutralizing autoantibodies production but a normal cellular response to type-I IFN could characterize high-risk individuals who might profit from IFN-Is treatment. Nevertheless, the advantages and disadvantages of this hypothesis and the best time for IFN-Is administration efficacy must be assessed precisely.

The major limitations of this study included failure to study other IFNs responses and fully investigate the phosphorylated form of proteins due to sample, temporal, and financial constraints and lack of IFN-I signaling and cytokine storm investigation due to alveolar sample collection restriction.

## Conclusion

Based on the study results, severe lymphopenia and high production of pro-inflammatory cytokines, reduced activity of IFN-I signaling molecules, and anti-IFN-I autoantibodies might be associated with the severity of COVID-19 disease. Despite appropriate progress in finding molecular mechanisms of coronavirus and developing more hopeful treatment medications and vaccines, future research should provide an accurate way to combat this virus.

## Data Availability

The data cannot be shared in public because of ethics and individual privacy restrictions but are limitedly available by contacting the corresponding author of this study, privately.

## Abbreviations

ELISA: Enzyme-linked immunosorbent assay
ARDS: Acute respiratory distress syndrome
CTLs: Cytotoxic CD8 + T lymphocytes
Th: T helper
PD-1: Programmed death 1
IL-6: Interleukin 6
TNF-α: Tumor necrosis factor-alpha
MODS: Multiple organ dysfunction syndromes
PRR: Pattern recognition receptors
MDA5: Melanoma differentiation-associated protein 5
RIG-I: Retinoic acid-inducible gene I
TLRs: Toll-like receptors
NF-κB: Nuclear factor-kappa B
GAF: γ-Interferon activating factor
ISGF3: IFN 3-stimulated gene factor
PID: Primary immune deficiencies
PMA: Phorbol myristate acetate
cDNA: Complementary DNA
PVDF: Polyvinylidene fluoride
BSA: Bovine serum albumin
ECL: Electrochemiluminescence
BMI: Body mass index
SBP: Systolic blood pressure
DBP: Diastolic blood pressure
HDL: High-density lipoprotein
GFR: Glomerular filtration rate
FBS: Fasting blood sugar
TG: Triglyceride
LDL: Low-density protein
CBC: Complete blood count
WBC: White blood cells
IFNs: Interferon's
STATs: Signal transducer and activator of transcriptions
JAK-1: Janus kinase 1
TYK-2: Tyrosine kinase 2
IRFs: The transcription factor interferon regulatory factors
Tregs: Regulatory T lymphocytes
NK cells: Natural killer cells
